# Evidence That the Heterogeneity of a T4 Population Is the Result of Heritable Traits

**DOI:** 10.1371/journal.pone.0116235

**Published:** 2014-12-31

**Authors:** Zachary J. Storms, Dominic Sauvageau

**Affiliations:** Department of Chemical and Materials Engineering, University of Alberta, Edmonton, Alberta, Canada; Rockefeller University, United States of America

## Abstract

Many bacteriophage populations display heterogeneity in their adsorption characteristics; a portion of the phage population remains free in solution throughout adsorption experiments (residual fraction). This residual fraction generally constitutes a minority of phages that exhibit significantly slower adsorption kinetics than the main phage stock (main fraction). While this phenomenon is likely the result of evolutionary driving forces, the present study demonstrates that the residual fraction is not always the result of phenotypic variations within a single genotype, as is generally thought. Experiments with phage T4 showed that two subgroups with distinct adsorption traits that were passed on to their progeny could be isolated from the original phage stock. Sequencing of genes involved in adsorption revealed two point mutations in gene 37 of residual fraction isolates, which resulted in modifications to the long tail-fiber, the organelle of attachment and host cell recognition. Adsorption studies consistently showed that T4 phage stocks amplified from residual fraction isolates had significantly lower adsorption efficiencies than those amplified from main fractions. The conducted experiments provide convincing evidence that the observed heterogeneity in T4 adsorption behavior is the result of conserved mutations to the phage genome and is not exclusively the result of phenotypic variations within the population. While it is believed high mutation rates exist to hasten phage adaptation, this study shows that this bet hedging strategy can also, in the short term, inadvertently handicap the phage's adsorption capabilities to a given host under normal infection conditions, resulting in the residual fraction observed in adsorption experiments.

## Introduction

Heterogeneity in the adsorption characteristics of a bacteriophage population has been widely reported since the first half of the 20^th^ century. Schlesinger [1], working with *Escherichia coli* phages, observed that a subset of the population had extremely slow adsorption kinetics or failed to adsorb at all. He called this group the residual fraction (summarized by Adams [1] and Delbrück [2]). Delbrück [2] confirmed these findings and ruled out the possibility of this fraction resulting from an equilibrium between phage and bacteria. This heterogeneity, whereby a fraction of the phage population either adsorbs extremely slowly or not at all in liquid media, has been reported in diverse tailed phage species. For example, “slow adsorbers” are known to account for 2–3% of the population of phage T1 under certain conditions [3], [4]. Similar observations have been noted for *Bacillus subtilis* phage PBS Z [5], *Lactobacillus casei* phage PL-1 [6], the flagella-targeting *Caulobacter crescentus* phage φCbK [7], various strains of phage λ [8], numerous T-series phages [9], [10] and even the mycoplasma virus L3 [11].

The practice of accounting for population heterogeneity in kinetic models of phage adsorption dates back to at least the work of Garen [4], who sometimes used smaller adsorption rate constants for the “slow adsorbing” fraction of a phage stock. This practice has been used by other researchers working with various phage-host systems [3], [6]. Some studies have even cautioned against the use of the two-step sequential model, a commonly used mechanism in which phage adsorption is modeled as a reversible interaction followed by an irreversible attachment [4], [12]–[18]. The heterogeneity of the phage population can misleadingly resemble the equilibrium between the free phage population and reversibly bound virus particles predicted by the sequential model [5], [10]. In actuality, it is more appropriate to model the system with a biphasic approach, where a portion of the phage population either binds very slowly or not at all [5], [9], [10]. Indeed, our recent studies have demonstrated how the inherent heterogeneity of T-series phage populations can be integrated into a model for phage adsorption that explicitly accounts for the non-adsorbing fraction of the phage population [9], [10].

While the existence of heterogeneous adsorption traits within a population has been demonstrated or inferred in many studies, its cause (or causes) has not been clearly elucidated. In fact, the heterogeneity of phage populations has been hypothesized to result from the presence of slow adsorbing virus particles [3], [4], [6], [8], [11], non-adsorbing phages [2], [5], [9], [10], [19] or adsorption efficiency [9], [10]. The adsorption efficiency is a term used to describe how readily a portion of a phage population adsorbs to a host cell in a specific set of conditions. The concepts of adsorption efficiency and residual fraction are easily reconciled by considering them as two different interpretations of the same phenomenon. Adsorption efficiency is defined in terms of the percentage of the population adsorbing under a certain set of conditions, while the residual fraction is defined as the percentage remaining free in solution. Previous studies have shown that the residual fraction (or adsorption efficiency) is strongly dependent on the physico-chemical properties of the medium, the physiology of the host cell, and the phenotype of the virus [8]–[10].

As previously mentioned, the reasons causing the biphasic adsorption behavior in phage populations are still not fully understood. The few studies that have examined the adsorption characteristics of specimens from the residual fraction have concluded that the traits are not heritable, as offspring displayed adsorption rates similar to the original population [8], [20]. A recent study on phage λ suggested that this feature may be an evolved form of diversified bet-hedging [21] to increase phenotypic diversity and therefore phage survival [8]. The existence of extremely slow adsorbing members of the population ensures some virus particles remain unattached during adverse growth conditions for the host. Experiments supported the hypothesis that the slow adsorbing phages comprising the residual fraction were the result of phenotypic variation within the population. Picking plaques formed by a residual phage and amplifying in a liquid culture resulted in a phage stock with traits characteristic of the original population. In addition, induction of lysogens formed from the residual fraction phages produced progeny exhibiting adsorption dynamics typical of the original stock. As an alternative to the bet-hedging hypothesis, the authors surmised that the slow adsorbing members of the population may suffer from a defective long tail-fiber (*Gp*J), the organelle of attachment, due to errors in protein assembly [8].

The present work shows that, unlike for phage λ, the heterogeneity found in populations of phage T4 is not caused by phenotypic variations within a single genotype but rather that the traits are the result of point mutations in the genes coding for the long tail-fibers of phage T4. The reasons for this heterogeneity are discussed in the context of the evolutionary struggle between virus and host. These findings show that more than one mechanism can explain the residual fraction found in different phage types.

## Materials and Methods

### Organisms and media

Bacterial cultures of *E. coli* ATCC 11303 were grown overnight in 25 ml of Bacto Tryptic Soy Broth (TSB; Becton, Dickinson and Company, Sparks, MD), agitated at 200 rpm and incubated at 37°C, to a concentration of ∼3×10^9^ cfu·ml^−1^. Phage T4 (ATCC 11303-B4), at a titer of 3×10^10^ pfu·ml^−1^, was stored at 4°C in TSB.

### Adsorption experiments

Adsorption experiments were carried out at room temperature in 1.5-ml microcentrifuge tubes using a stationary phase bacterial culture to prevent productive infections leading to cell lysis. A 1.0-ml sample of overnight culture broth was mixed with 10 µl of phage solution, adjusted for a final multiplicity of infection (MOI) of 0.1–0.5. At intervals, samples were removed, diluted in 1.0 ml of fresh medium, and centrifuged for 1 minute at 14,000×g. The supernatant, containing free (unadsorbed) phage particles, was then immediately collected and plated, after appropriate dilution, on a TSB-agar plate using a modified version of the agar layering technique described previously [9]. *E. coli* ATCC 11303 was used as the indicator strain. Plates were incubated for at least 12 hours at 37°C and titers were determined as an average of duplicate assays. Adsorption data is presented as a decrease in the free phage fraction, defined as the free phage titer divided by the initial phage titer.

It should be noted that the technique used to gather the adsorption data presented here does not lend itself to reliable kinetic data and that this was not the aim of the present study. Reaction kinetics are very fast for phage T4 at the elevated phage and cell concentrations used in the experiments [9]. Therefore, the time required to isolate a sample introduces significant error to the determination of a reaction rate constant (*k*-value). Consequently, reaction rate constants were not determined and are not reported in this study. Other researchers have commented on this drawback [8]. For reliable kinetic data, it is necessary to either change the experimental conditions to slow down the adsorption process (i.e. lower temperature, dilute cell/phage concentration) or use an alternative technique, such as filtering the samples immediately at the desired time intervals (see the protocol reported in [9]).

### Isolation of two distinct subgroups

The heterogeneity of the phage population was studied by dividing the original stock solution into two subgroups: the main fraction and the residual fraction. The main fraction represents the vast majority of the population, roughly 97% in the conditions tested, which adsorbed to a host cell within 2–3 minutes under the conditions of the adsorption experiment. The main fraction was isolated by removing a 100-µl sample after 30 seconds of adsorption, centrifuging for 1 minute at 14,000×g, and then re-suspending the pellet, which contained cells infected by the main fraction, in 1.0 ml of fresh media. This suspension was then transferred to 25 ml of pre-warmed TSB in a shake flask and incubated overnight at 200 rpm and 37°C to amplify the phage stock. After amplification, phages were isolated by filtering the culture through a 0.2 µm syringe filter.

The residual fraction is defined, in this context, as those phages unable to adsorb to a host cell over the course of the adsorption experiment. It should be noted that the free phage fraction remained generally unchanged after the first 5 minutes of adsorption, as the adsorption rate approached zero. Consequently, the residual fraction of the phage population was isolated and amplified by centrifuging the entire infection volume after 30 minutes of adsorption for 1 minute at 14,000×g and mixing a 350 µl aliquot of the supernatant, containing free (unadsorbed) phages, with 350 µl of overnight culture. The mixture was transferred to 25 ml of pre-warmed TSB in a shake flask and incubated overnight at 200 rpm and 37°C to amplify the phage stock. After amplification, phages were isolated by filtering the culture through a 0.2 µm syringe filter. These isolation/amplification steps were performed in series to obtain multiple generations of a given fraction. In some instances, individual plaques were picked and amplified to study the inheritability of observable traits.

### Phage amplification

Amplification dynamics of some phage stocks were studied in detail. For these experiments, 500 µl of early log phase *E. coli* cultures (10^8^ cfu·ml^−1^) were mixed with 10 µl of phage stock to achieve an initial MOI of 0.001. After 5 minutes to allow for adsorption, the mixture was transferred to 25 ml of pre-warmed TSB in a 250 ml shake flask. Cultures were incubated at 37°C and agitated at 200 rpm for 3 hours. Samples were removed hourly to measure optical density and assay for mature phages.

### DNA sequencing

Samples of selected phage stocks were sequenced at the Molecular Biology Services Unit of the University of Alberta with a BigDye Terminator v3.1 Cycle Sequencing Kit from Applied Biosystems (Foster City, CA). Phage DNA was first isolated using a phage DNA isolation kit (Norgen Biotek, Ottawa, ON) according to manufacturer's instructions. Next, a 3,840 bp DNA fragment containing genes 37 and 38 was PCR amplified from each sample using Q5 high fidelity polymerase (New England Biolabs, Ipswich, MA) in a Bio-Rad T100 Thermal Cycler (34 cycles of 98°C for 10 s, 50°C for 30 s, 72°C for 2 min) and purified with a QIAquick PCR Purification Kit (Qiagen, Toronto, ON). Using 5 primers initiated at different nucleotides of the amplified fragment (see Table 1), genes 37 and 38 were fluorescently labelled with dideoxynucleotides following the protocol of Applied Biosystems for BigDye Terminator v3.1, purified by ethanol precipitation, and analyzed in a ABI 3730 DNA analyzer. The sequencing process was completed with at least 2x coverage for each stock in order to verify mutations.

**Table 1:** Primers used in PCR amplification and sequencing.

Primer^1^	Sense	Sequence (5′–3′)	Application
**G36-578**	Forward	CGCAGAATCCTGCATCTCAA	Amplification/Sequencing
**G37-740**	Forward	TTTCTTGGTGGTCTGGTGATAC	Sequencing
**G37-1412**	Forward	GTACTCGCGAAGGACAGAATAAA	Sequencing
**G37-2665** ^2^	Forward	GCTTCAAGTACTGACTTAGG	Sequencing
**GT-65**	Reverse	ACAAGCGATCTAGAACACCAAATA	Amplification/Sequencing

1 Primer name consists of gene and nucleotide start position on that gene. ^2^Codes for the hypervariable region identified by Tetart *et al.* [32].

## Results

### Nomenclature and experimental design

**Figure 1:**
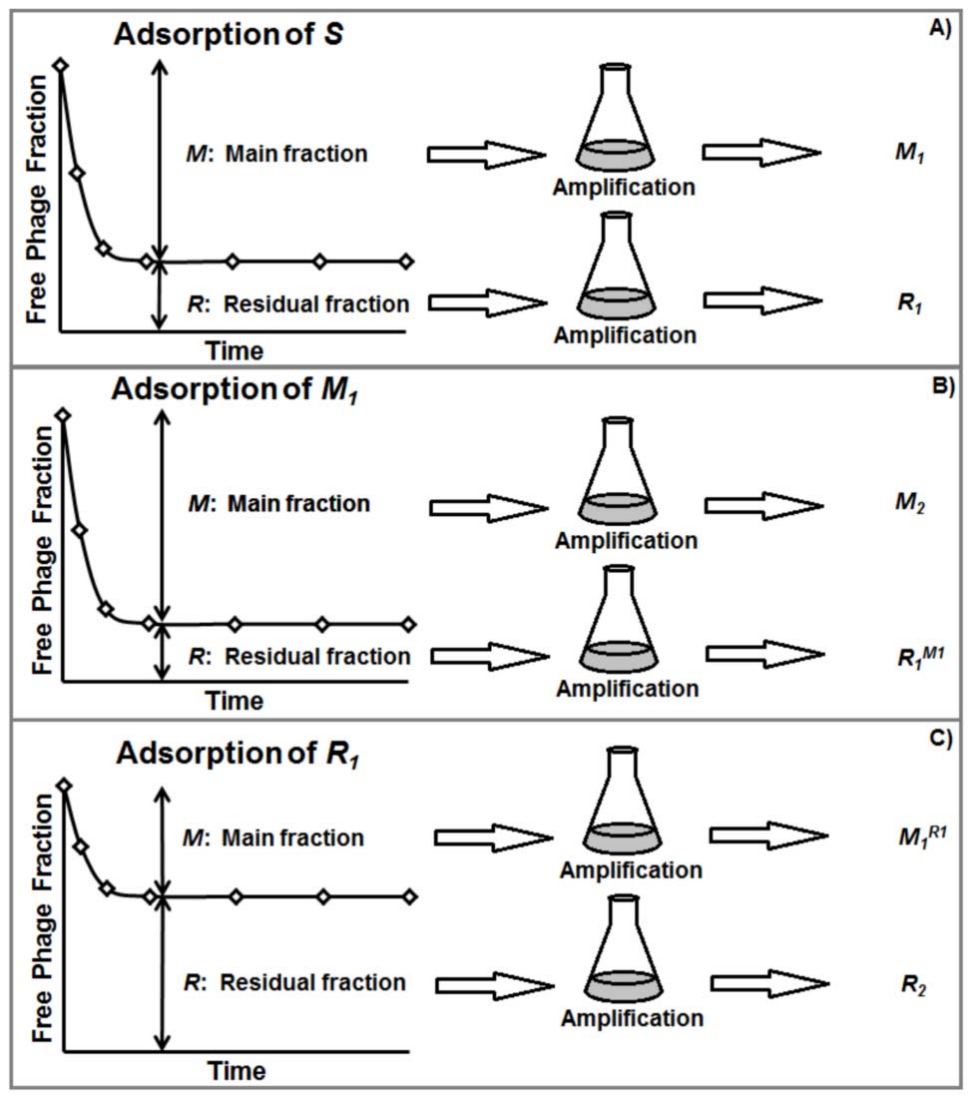
Schematic of adsorption experiments and nomenclature. A) Adsorption of the original phage stock *S*, used to isolate the residual fraction *R_1_* – phages that did not adsorb – and the main fraction *M_1_* – phages that adsorbed. B) Adsorption of the *M_1_* subgroup, used to isolate the main fraction *M_2_* and the residual from a main fraction isolate *R_1_^M1^*. C) Adsorption of the *R_1_* subgroup, used to isolate the residual fraction *R_2_* and main fraction from a residual fraction isolate *M_1_^R1^*.

The following nomenclature is introduced to describe different sets of phage T4 subgroups presented in this study (refer to Fig. 1 for a map of experiments, samples and related nomenclature). The original T4 stock solution is denoted as *S*. From *S*, a residual fraction *R_1_* (unadsorbed phages) and main fraction *M_1_* (adsorbed phages) were isolated and amplified as described above (Fig. 1A). From each of these new subgroups, a residual and main fraction could also be isolated. The main fraction obtained from the adsorption of an *M_1_* sample is called *M_2_* and its residual, *R_1_^M1^* (Fig. 1B); the residual fraction of *R_1_* is called *R_2_* and its main fraction *M_1_^R1^* (Fig. 1C), and so on. For the main fractions, which consists of phages that readily bind to and infect the cells in liquid culture, it was always necessary to amplify the culture in order to isolate the phage subgroup. However, the residual fractions could be isolated without amplification from the supernatant of the centrifuged infection broth. Experiments on this unamplified residual fraction confirmed that members of this group had substantially slower rates of adsorption than the original phage stock, an observation widely reported by other researchers [1], [2], [8], [20] and not discussed here. Instead, the focus of this study was to evaluate the hypothesis that adsorption traits of viruses from the residual fraction are conserved. In addition, individual plaques were isolated, amplified, and analyzed. This is denoted by adding *P_i_* to the stock symbol where the subscript refers to the *i^th^* amplification performed on the plaque. For example, *M_8_P_1_* denotes a phage population amplified once starting from a single plaque of the *M_8_* stock. A population amplified from a single plaque of *M_8_P_1_* would be called *M_8_P_2_*, and so on.

### Adsorption characteristics of the isolated phage stocks

**Figure 2:**
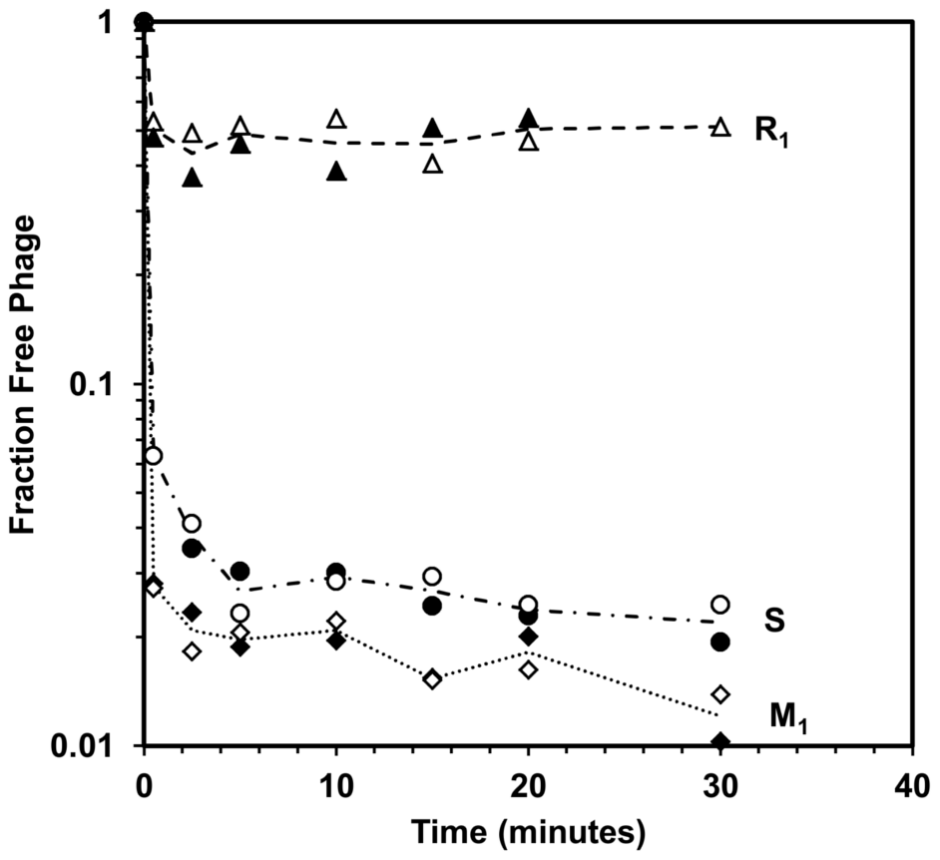
Adsorption of the T4 main and residual fractions. The main fraction *M_1_* (diamonds), and residual fraction *R_1_* (triangles), were isolated and amplified from the same T4 stock *S* (circles). The number of free phages, plotted as a fraction of the initial titer, from adsorption experiments in TSB using stationary phase *E. coli* cultures is shown. Solid and open symbols represent replicate experiments. All experiments were carried out at 24°C and an initial MOI of ∼0.1 with a host cell concentration of ∼3×10^9^ cfu·ml^−1^. The curves indicate the trends of two replicates and are not the result of a modeling equation.

The heterogeneity of the T4 population is prominently displayed in the adsorption data presented in Fig. 2. Isolation and amplification of the main and residual fractions resulted in adsorption behaviors distinct from the original T4 stock (*S*). The *M_1_* subgroup – efficiently adsorbing phages – had a smaller residual fraction than *S*, indicating an improvement in adsorption efficiency. On the other hand, the *R_1_* subgroup – stemming from phages that did not adsorb – had a considerably larger residual fraction than *S* (Fig. 2). Further rounds of isolation/amplification of *R_1_* or *M_1_* did not change the adsorption behavior. Both the *M_1_* and *R_1_* subgroups displayed very fast adsorption kinetics, followed by a long period of no discernible adsorption. The significant difference between the subgroups (*R_i_* and *M_i_*) lay in the adsorption efficiency, defined as the percentage of the phage population able to adsorb before the free phage fraction reached a plateau. For the main fraction (*M_1_*), this value was 98% or more; for the residual fraction (*R_1_*), it was slightly above 50%.

In general terms, the phage stocks tested in this study exhibited one of two types of adsorption efficiency. The first set, which can generally be referred to as *M*-type phages – or efficiently adsorbing phages – was characterized by fast kinetics and a high adsorption efficiency (>97%). The second set, which will be referred to as *R*-type phages, also had fast kinetics but significantly lower adsorption efficiency (40–80%).

### Plaque morphology

**Figure 3:**
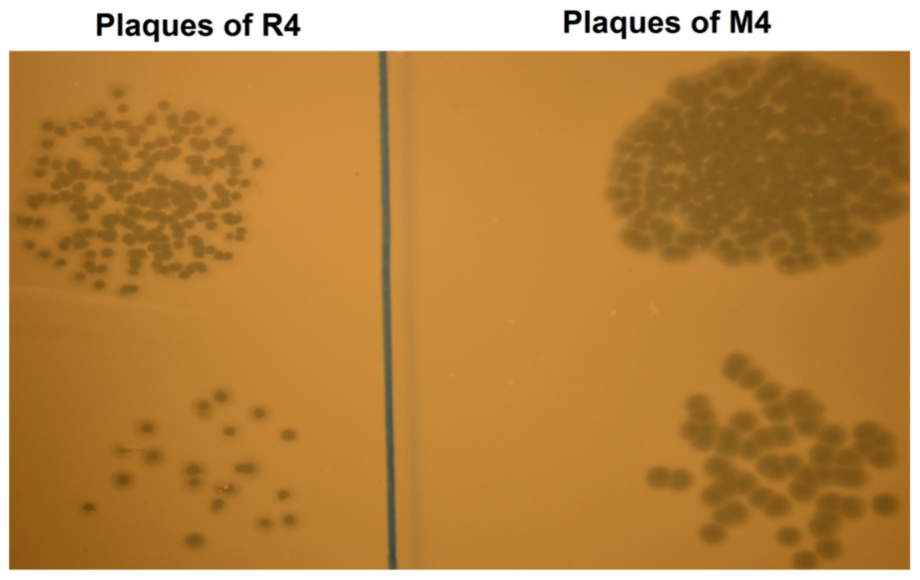
Plaque morphology. The plaque morphologies of the *R_4_* and *M_4_* phage stocks. The left side of the picture depicts plaques formed from the *R_4_* subgroup. The right side of the picture depicts plaques formed by the *M_4_* subgroup.

While the residual fraction consisted of phages with a very poor aptitude for adsorption in liquid culture, they were still able to form plaques on an agar plate. Indeed, measuring the titer of the residual phages is how one determines the free phage fraction reported in the adsorption data presented here and elsewhere [8]. However, Fig. 3 demonstrates the distinct plaque morphologies characteristic of the *M_i_* and *R_i_* subgroups. The plaques formed by members of the residual fraction (*R_4_* is shown) were significantly smaller than the large plaques of the *M_4_* subgroup, consistent with observations reported for the residual fraction of phage λ [8].

### Dynamics of infection differ between main fraction and residual fraction phages

**Figure 4:**
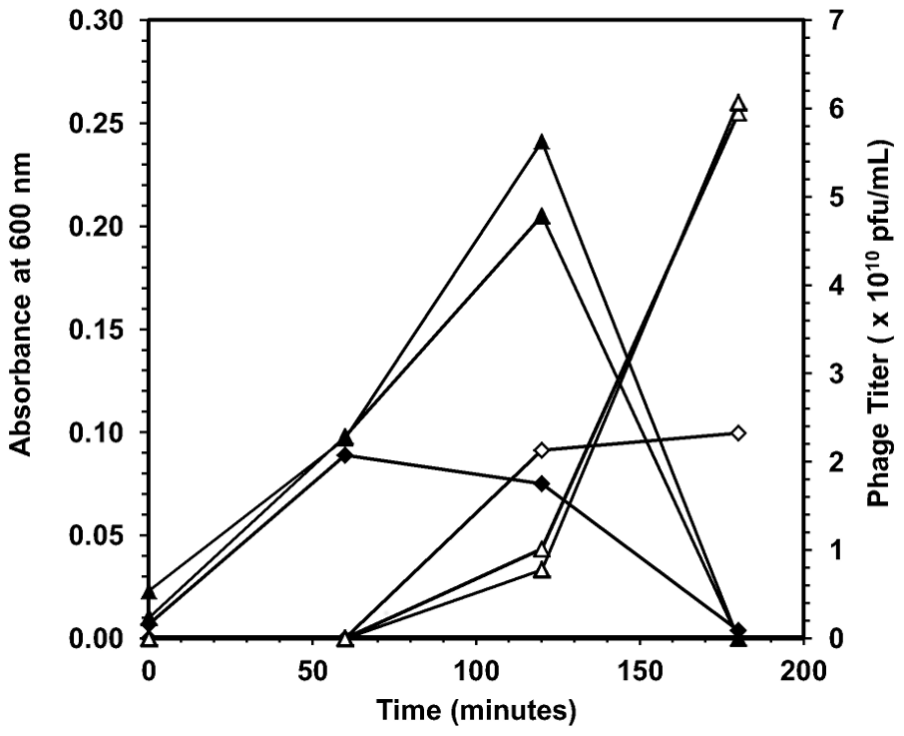
Amplification of the main and residual fractions. Propagation of the *M_1_* (diamonds) and *R_1_* (triangles) phage stocks in TSB. The phage titer is shown as open symbols and optical density is shown as closed symbols. *E. coli* cultures from the early log phase of growth (10^8^ cfu·ml^−1^) were mixed with phage solutions in 0.8 ml of broth at an MOI of 0.001. After allowing 5 minutes for adsorption, the mixture was transferred to 25 ml of pre-warmed TSB in a 250-ml shake flask, incubated at 37°C, and aerated at 200 rpm for 3 hours. The *R_1_* experiment was performed in duplicate.

While the adsorption curves of Fig. 2 indicate that the phages of the residual fraction adsorb poorly in liquid culture, they are still able to propagate quite easily under the appropriate conditions (Fig. 4). The host receptors of phage T4, cell lipopolysaccharides and outer membrane protein C [22], are expressed through all stages of cell growth. Receptor expression level is not expected to impact phage amplification under the conditions of the experiment. At an initial MOI of 0.001, the residual stock *R_1_* produced over 2.5 times more phages than the *M_1_* subgroup. The *M_1_* subgroup reached a maximum titer within 2 hours with a higher specific productivity (1.36×10^9^ pfu·ml^−1^·OD^−1^·h^−1^) than the *R_1_* subgroups (1.02×10^9^ pfu·ml^−1^·OD^−1^·h^−1^), but at the expense of a lower final phage titer. The low adsorption efficiency of the *R_1_* subgroup resulted in a longer production time, which allowed for more bacterial growth (see optical density curves of Fig. 4), and consequently a larger final phage titer. These observations are consistent with similar batch production studies on T4 where the adsorption efficiency was varied using the amino acid L-tryptophan [9].

### Characterization of the heterogeneity of phage T4 populations

**Figure 5:**
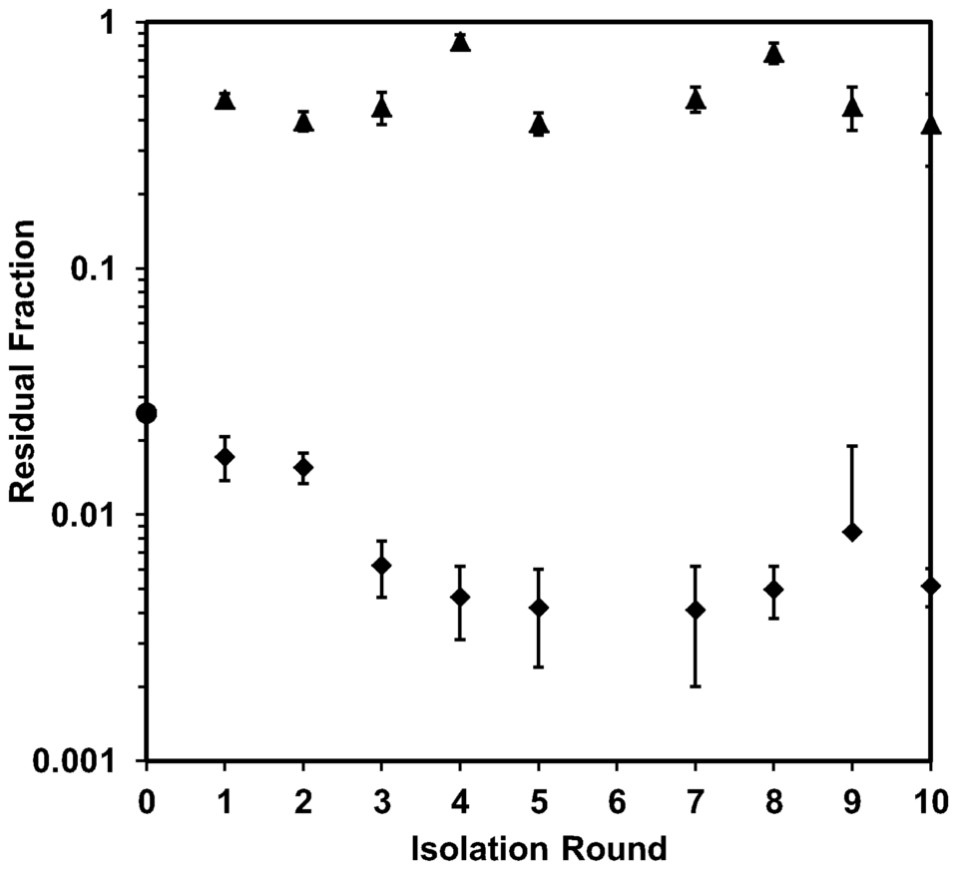
Residual fractions of the *M*-type and *R*-type phage stocks. The value of the residual fraction, reported as a fraction of the initial phage titer, from adsorption experiments performed with phage stocks *M_1_* through *M_10_* (diamonds) and *R_1_* through *R_10_* (triangles). The abscissa shows the *i^th^* isolation round (i.e. *M_3_* is plotted at point 3). The residual of *S* (circle) is shown as a reference at point 0. Each value is calculated as the average of five points collected during an adsorption experiment. Errors bars show the standard deviation.

The nature of the heterogeneity of the T4 population was studied through a series of isolation and amplification experiments. The residual fraction from the adsorption of the *R_1_* subgroup (labeled *R_2_*) and the main fraction from the adsorption of the *M_1_* subgroup (labeled *M_2_*) were isolated and amplified. Individual adsorption experiments were performed using the *R_2_* and *M_2_* subgroups, from which the *R_3_* and *M_3_* subgroups were respectively isolated. This process was completed for 10 rounds of isolation and amplification for each fraction. The results are summarized in Fig. 5. The abscissa indicates the isolation round while the ordinate shows the magnitude of the residual fraction determined for each subgroup. Round 0 represents the residual fraction of the original phage stock (*S*). First, it should be noted that the fast adsorption kinetics displayed in Fig. 2 were observed for all stocks of the *R_i_* and *M_i_* subgroups. The *R_i_* series did not see significant change in the adsorption curve from one isolate to the next; the average residual fraction (with standard deviation) for all *R_i_* series subgroups remained stable at 0.51±0.16. However, the residual fraction decreased in the *M_i_* series during the first three isolation rounds before stabilizing at 0.005±0.0015, a full two orders of magnitude lower than the *R_i_* series average residual fraction. Despite the difference in magnitude, coefficients of variance of the residual fraction for the *R_i_* series and *M_i_* series were very similar (0.31 and 0.30, respectively).

The results of the isolation/amplification experiments with the *M_i_* series revealed that, despite selecting for efficiently adsorbing phages, 1) all phage populations had a residual fraction and 2) after a small decrease, the value of this fraction remained constant. Indeed, amplification of the residual fraction of a *M_i_* subgroup (the *R_1_^Mi^* subgroups) yielded phage populations with adsorption behavior similar to the *R_i_* subgroups (S1 Fig.). While it was possible to isolate *R*-type phages from the *M_i_* subgroups, it was not possible to isolate efficiently adsorbing phages from the *R_i_* subgroups. Amplification of the main fraction of a *R_i_* subgroup (the *M_1_^Ri^* subgroups) did not yield phages with the high adsorption efficiencies typical of the *M_i_* subgroups (S1 Fig.). Like the *R_1_^Mi^* subgroups, the *M_1_^Ri^* subgroups exhibited low adsorption efficiencies typical of *R_i_* subgroups, being consistent with the poor adsorption being a heritable trait.

**Figure 6:**
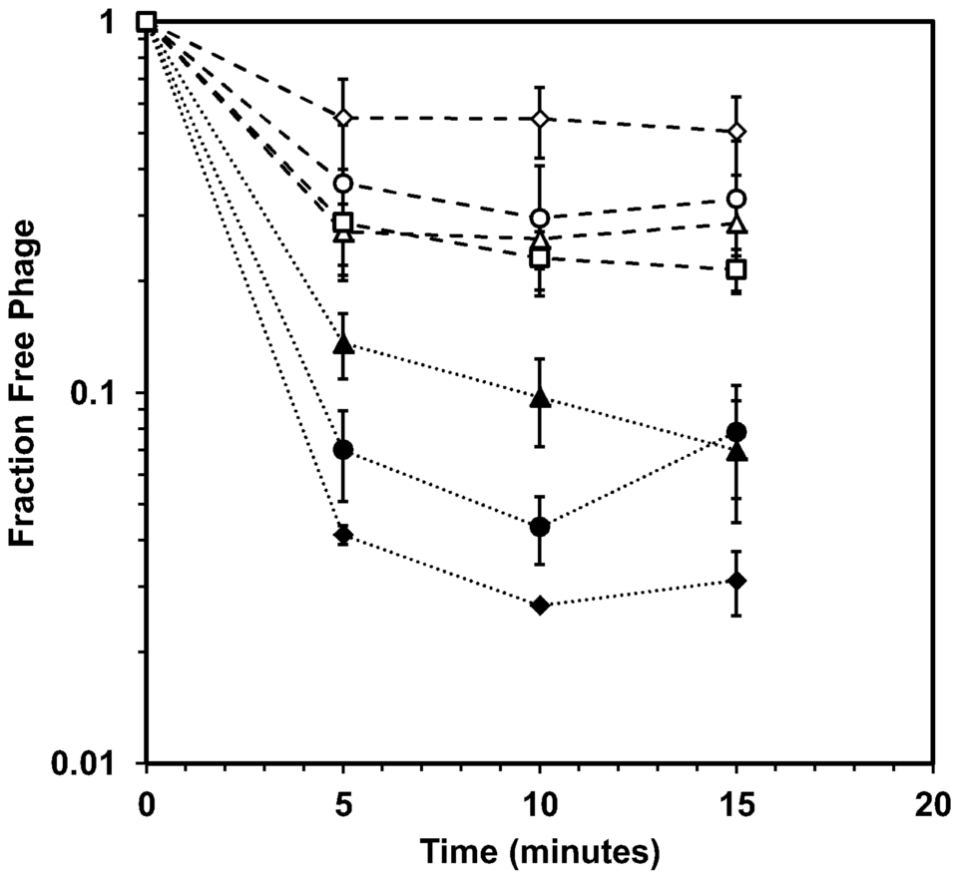
Adsorption of populations originating from single plaques. Adsorption curves of of *R*-type and *M*-type phage stocks isolated from individual plaques of the *R_8_* and *M_8_* populations are compared: *R_8_* (open diamonds), *R_8_P_1_* (open triangles), *R_8_P_2_* (open circles), *M_8_* (closed diamonds), *M_8_P_1_* (closed triangles), *M_8_P_2_* (closed circles), and the plaque isolated residuals of *M_8_P_1_* (open squares). Error bars represent the standard deviation of three replicates for *R_8_* and *M_8_*. For the plaque-isolated cultures, error bars present the standard deviation of at least three individual plaque-isolated cultures.

In order to test whether the heterogeneity of the population was due to the presence of an initial *R*-mutant in the population, individual plaques of stocks *M_8_* and *R_8_* were isolated and amplified. Five plaques were picked from each stock and amplified overnight. From these new populations, an additional 6 plaques were picked, 3 from the *M_8_P_1_* stocks and 3 from the *R_8_P_1_* stocks. The adsorption curves of these plaque-isolated cultures are summarized in Fig. 6. The average residual fractions for *R_8_*, *R_8_P_1_*, and *R_8_P_2_*, were 0.53±0.11, 0.27±0.05, and 0.35±0.15, respectively. The average residual fractions for *M_8_*, *M_8_P_1_*, and *M_8_P_2_*, were 0.033±0.007, 0.10±0.037, and 0.064±0.023, respectively. It should be noted that these experiments were conducted after the *M_8_* and *R_8_* stocks had been stored at 4°C for an extended period of time during which both stocks suffered a 10-fold decrease in titer due to natural deactivation. The deactivated phages would appear to have been of the *M*-type, since there was a corresponding 10-fold increase in the size of the residual fraction for *M_8_* (compare Figs. 6 and 5). While variation among the *R*-type stocks is evident, amplifying multiple generations of plaques of the *R_8_* stock did not yield a significant change in the residual fraction (0.35 vs 0.53, Student's t-test, p>0.05). Similarly, multiple rounds of plaque isolation and amplification of the *M_8_* stock yielded a phage population with a residual fraction 9 times smaller than the *R_8_* stock (0.064 vs. 0.53). Finally, amplification of three residual plaques isolated from the *M_8_P_1_* samples yielded phage stocks with an average residual fraction of 0.24±0.06 (Fig. 6), statistically equal to both the *R_8_P_1_*, and *R_8_P_2_* stocks (Student's t-test, p>0.05).

### Genetic analysis

**Figure 7:**
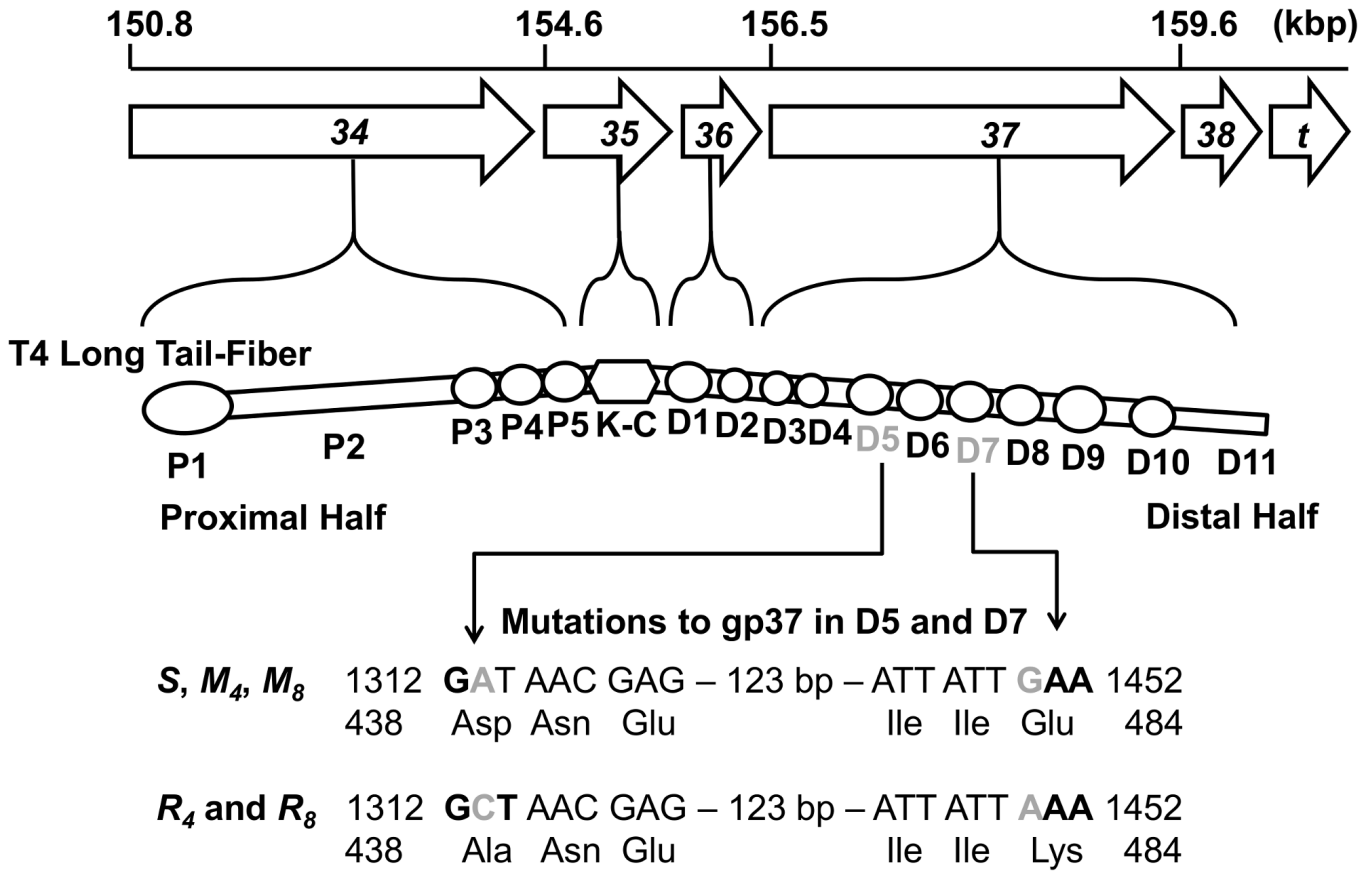
Point mutations found in gene 37 of the residual stocks. Genes 34, 35, 36, and 37 code for the major structural proteins of the phage T4 long tail-fiber (diagram of the long tail-fiber structure is adapted from Fig. 1 of [38]). The residual stocks contained two point mutations resulting in changes to the amino acid sequence of domains D5 and D7 of the long tail-fiber.

DNA sequencing revealed genetic differences between the *R*-type and *M*-type phage stocks in gene 37, which codes for the protein constituting the distal half of the phage T4 long tail-fiber [23] (Fig. 7). The phage T4 long tail-fiber is the organelle of attachment and recognition [24]. Gene 38 was also sequenced since proper trimeric assembly of gp37 is assured with the assistance of gp38 [25]. Two residual fraction stocks (*R_4_* and *R_8_*) and two main fraction stocks (*M_4_* and *M_8_*) were selected for partial DNA sequencing. Using BLAST analysis (ncbi.nlm.nih.gov), the nucleotide sequences of genes 37 and 38 from these stocks were compared to corresponding sequences of the parent wild-type T4 stock (*S*) and of T4 strains available on the NCBI database.

The parent T4 stock is a wild-type strain that aligned nearly completely in genes 37 and 38 with wild-type phage T4T except for two nucleotides (positions 1,250 and 1,450 of gene 37). The main fraction stocks *M_4_* and *M_8_* contained the exact nucleotide sequence of the parent T4 stock *S* in both genes 37 and 38; the residual fraction stocks, *R_4_* and *R_8_*, contained two important differences. While gene 38 of *R_4_* and *R_8_* aligned completely with gene 38 of *S*, gene 37 contained two distinct point mutations which resulted in new amino acid residues on gp37 (Fig. 7). The substitution of adenine (A) with cytosine (C) at nucleotide 1,313 replaced a negatively charged aspartic acid (Asp) at residue 438 of the wild-type T4 with a nonpolar alanine (Ala). At nucleotide 1,450 of *R_4_* and *R_8_*, guanine (G) was substituted by adenine (A), resulting in a positively charged lysine (Lys) supplanting the negatively charged glutamic acid (Glu) at residue 484 of the wild-type strain. Residues 438 and 484 are found in domains D5 and D7, respectively, of the distal half of the T4 long-tail fiber [23] (Fig. 7).

## Discussion

A recent study on strains of phage λ with varying adsorption rates concluded that the residual fraction was not due to genotypic variation because characteristics of the residual phages were not passed on to their progeny [8]. The authors suggested that the existence of the residual fraction may have evolved as a form of diversified bet-hedging [21]. According to this theory, the slow adsorbing members of the population would be the result of phenotypic variations of the same genotype, evolved to guarantee some phages remain unadsorbed during periods of poor growth conditions that inhibit productive infection of the host cell (while some phages such as T7 can replicate in a stationary phase culture, most cannot). This can be thought of as an alternative strategy to lysogeny or pseudolysogeny, which are common tactics employed by bacterial viruses to survive long periods of nutrient limitation [26]–[28]. Another proposed explanation by the authors for the existence of a residual fraction is errors in protein processing [8], which can be as high as 0.1% in biological systems [29]. Transcription and translation errors may even comprise an evolutionary advantage in the development of complex mutations [29], [30]. At least two lactococcal phages have been shown to exhibit heterogeneity in their adsorption characteristics due to proteolytic processing of tail fiber proteins [31]. Regardless of the explanation, the hypothesis that the heterogeneity of phage populations provides an evolutionary advantage to the virus is quite plausible and can explain residual fractions in many phage-host systems.

Adsorption data reported for a wide range of tailed phage families all suggest the existence of a residual fraction [1]–[11], [19], [20]. Therefore, it is possible that the heterogeneity of a phage stock is a universal phenomenon common to most or even all phages. It seems likely that phages have developed different strategies (related to phenotypic and/or genotypic variations) to seek the evolutionary advantage of adsorption heterogeneity. While the work of Gallet *et al.* [8] supports the hypothesis that phenotypic stochasticity or malfunctions in protein assembly explains the heterogeneity of λ cultures, the present study provides evidence that the heterogeneity of a phage T4 population is instead caused by genotypic diversity within the same population.

Should the adsorption trait be due to phenotypic variations of a specific T4 genotype, one would expect that the poorly adsorbing phage trait would be superseded by the efficiently adsorbing phages during the amplification process. According to the diversified bet-hedging theory, phage particles displaying the slow adsorbing phenotype would be expected to produce *M*-type and *R*-type progeny in the same ratio as those displaying the fast adsorbing phenotype [21], a phenomenon observed in phage λ [8] but not in phage T4 (Figs. 4, 5 and 6). Similarly, if the adsorption traits were brought about by errors in protein processing, isolated phages from the residual fraction should produce progeny with adsorption behavior equivalent to that of the original stock *S*. In actuality, this did not occur, even when isolating individual plaques (Fig. 6). Instead, the poor adsorption efficiency of the residual fraction was passed on to progeny phages during amplification (Figs. 5 and 6). The heritability of the adsorption characteristics of *R_1_*, which was isolated from the original stock (*S*), thus suggests at least some modifications to the phage genome are at work and passed on to further generations in a phage population.

Importantly, the heterogeneity of the phage population is not merely the result of *R*-mutants already present in the phage population. Attempts were made to isolate “pure” *M*-type phages from *M_8_* plaques. All plaques isolated in this way produced phage populations with observable residual fractions (*M_8_P_1_* and *M_8_P_2_*, Fig. 6). Furthermore, isolation and amplification of plaques taken from the residual group of these “pure” stocks yielded *de novo R*-type populations with poor adsorption efficiency (residual of *M_8_P_1_*, Fig. 6). If these phages were adsorption delinquent merely due to random transcriptional or translational errors, they would have produced progeny with the same adsorption traits as the parent strain. The results of Fig. 6 suggest that even within the small number of generations needed to amplify a single phage to a working stock, phage T4 mutates into distinct groups with unique adsorption characteristics.

The adsorption characteristics of *R*-type and *M*-type phages were not the only inherited traits observed in the study. The poor adsorption efficiency of the *R*-type phages in liquid culture translated to slow plaque development on agar plates (Fig. 3) and lower phage productivity rates during amplification in liquid cultures (Fig. 4). While the residual fraction consists of phages that are unable to adsorb to a host cell over the course of an adsorption experiment, clearly they are not completely deficient as they have the ability to form plaques on agar lawns. In all likelihood, the residual fraction itself consists of a heterogeneous population with a distribution of adsorption rates and/or efficiencies that are significantly lower than the adsorption rate and/or efficiency of the main fraction phages; a hypothesis supported by other studies [8]. Stocks amplified from individual plaques of *R*-type phages yielded plaques of similar morphology to those shown in Fig. 3, providing further evidence that the adsorption traits are conserved from one generation to the next.

The reduction in phage productivity observed for *R*-type phages during stock amplification (Fig. 4) is expected only if the low adsorption efficiency of the residual fraction represents a heritable trait. It is a well understood phenomenon in phage ecology that lowering the initial MOI in an amplification experiment results in a longer growth period for the host cells, usually resulting in higher final titers. This is due to the fact that the reduction in the initial number of virus particles allows the cell culture to grow to a higher density before the phage overtakes the population and population-wide lysis is observed. The authors have previously shown that reducing the adsorption efficiency of a phage stock through the manipulation of environmental parameters has a similar effect to lowering the initial MOI in an amplification experiment [9]. Comparison of the amplification dynamics of the *M_1_* and *R_1_* phage stocks shown in Fig. 4 reveals that the *R_1_* phages indeed behave as if the initial MOI had been reduced, as evidenced by both the longer period of cell growth before population-wide lysis and the higher final titer obtained. The dynamics of infection were greatly modified by simply using a phage stock with inherently low adsorption efficiency – a phenomenon that normally requires environmental manipulation – providing another validation of the assumption that the poor adsorption efficiency of the *R*-type phages is a conserved trait passed on to progeny.

There are many possible genetic causes for the poorly adsorbing behavior observed in the residual fraction. For example, mutations to any number of genes coding for phage proteins could lead to adverse structural or morphological changes to the mature phage particle. In this study, DNA sequencing focused on genes coding for what were considered likely candidate proteins influencing adsorption capability: the long tail fibers. The phage T4 long tail-fiber, as shown in Fig. 7, is the organelle of attachment and recognition [24]. The distal half, which interacts directly with the host cell, is composed of a trimer of gp37 [23]. Proper trimeric assembly of gp37 is assured by the product of gene 38 [25]. Consequently, mutations to genes 37 and 38 are most likely to directly affect the adsorption behavior of the virus.

The results of DNA sequencing highlighted two mutations in gene 37 of the *R_4_* and *R_8_* stocks that offer the most comprehensive explanation for the differences in adsorption behavior among the phage stocks tested (Fig. 7). Located in domains D5 and D7 of the distal half of the T4 long tail-fiber, these mutations introduce amino acid residues with significant differences in their side chain structure and charge. The distribution of polar and nonpolar side chains is an important factor governing the folding of a protein. Therefore, disrupting the charge balance on these important structural globules could potentially lead to a weakened or dysfunctional tail fiber. For example, in D5, a hydrophobic, nonpolar molecule (Ala) replaces a negatively charged hydrophilic molecule (Asp). Such a shift in chemical characteristics is almost certain to impact the structure and/or the receptor affinity of the long tail-fiber. The fact that both the *R_4_* and *R_8_* stocks possessed the same genetic defects provides conclusive evidence that point mutations are conserved from one generation to the next (Fig. 7).

It should be noted that samples for DNA sequencing were taken from amplified stocks, rather than from single plaques. This may seem, at first, an unconventional practice. It does not enable the identification of the full genotypic variations present in the stock. It will only allow the determination of the sequence of the predominant genotype present, as opposed to the genotype of a specific phage particle. However, considering the implications of the results presented here and that even a single plaque is the product of multiple rounds of progeny, samples taken from any stock solution or any plaque will contain a variety of genotypes. This is supported by the results of Fig. 6, where it was possible to isolate populations with distinct adsorption behavior derived from a single, common ancestor. In fact, a T4 plaque likely consists of millions of phage particles propagated over multiple generations. Therefore, the DNA sequence obtained from any sample will be representative of the predominant genotype within that plaque or stock. The sampling method for DNA sequencing in no way diminishes the fact that the residual fraction phages isolated did, in fact, contain two conserved point mutations to gene 37.

Is the existence of two point mutations in the long tail-fiber of a phage T4 mutant enough to explain the drastic changes in adsorption behavior among the *R*-type and *M*-type populations? The results of similar studies suggest that it is. For example, one study on phage T4 found that it could switch hosts from *E. coli* to *Yersinia pseudotuberculosis* with the substitution of a single amino acid residue in gene 37 [32]. Similarly, a single point mutation in phage ΦEF24C, believed to affect the tail-fiber, has been reported to significantly improve adsorption to *Enterococcus faecalis* [33]. Finally, a comprehensive analysis of the T2-like phage Ox2 found that it contained specific, hypervariable regions in gene 38 which enabled the virus to expand its host receptor specificity to include various outer membrane proteins [34], [35]. In fact, some mutants of Ox2 were able to switch from a protein receptor to a carbohydrate [34].

Most studies have suggested that fast mutation rates within phage species can lead to broader host ranges or more efficient adsorption. Some have even speculated that the high rates of mutation observed are a direct result of the Darwinian struggle between predator and prey [34]. A common method to shield a cell from virus invasion is to prevent the very first step in the process: attachment to the host [36], [37]. Changes in the receptors used for the reversible or irreversible step in virus adsorption can lead to resistant bacterial strains. Consequently, evolutionary driving forces may have placed pressure on the regions within some phage genomes that code for the phage adsorption machinery to be more prone to mutations. Such “hyper-variable” regions have been identified for T4 [32] and T4-like phages [34]. A side-effect of this variability is the incidental production of phage particles with poor adsorption capabilities to the specific host being studied, leading to the residual fraction observed in most phage T4 adsorption experiments. It may thus be tempting to consider the residual fraction of phage T4 a by-product of evolution; engineering mishaps in the race for improved phage fitness; however, the more complete view sees the potential benefits of poorly adsorbing members of the phage population. As noted by Gallet *et al.* [8], the residual fraction assures some phages remain free in solution through long periods of adverse host growth conditions, helping to ensure phage survival through times of hardship. In addition, the adsorption efficiency of a population has been shown to strongly correlate with the conditions of infection [9], [10], suggesting the residual fraction contains phages able to adsorb well in a different environment.

The results presented in this study provide strong evidence that the residual fraction of phage T4, unlike that of other phages such as λ, stems, at least partially, from a conserved trait passed on to progeny. Residual fraction phages consistently demonstrated poor adsorption efficiencies, small plaque morphology, and lower amplification productivities. Numerous rounds of amplification could not yield phage populations characteristic of the parent strain. Even single plaques of *M*-type phages produced small numbers of *R*-type phages whose adsorption traits were both distinct from the parent strain and passed on to progeny. The best explanation for the differences between the residual fraction phages and the main fraction phages observed in this study is the presence of two point mutations in gene 37, which are likely to impact the structure of the long tail-fiber used in host cell attachment and recognition. The results suggest further experimentation would lead to additional mutations that could either hinder or improve phage adsorption. These small defects, while generally detrimental to phage fitness in the experiments performed, might represent an overall, long-term evolutionary benefit by assuring some phage particles remain free in solution during adverse growth conditions. These mutations can also explain the residual fraction observed in phage T4.

## Supporting Information

S1 Fig **Adsorption of** ***R_1_^Mi^*** **and** ***M_1_^Ri^*** **phage stocks.** A) The adsorption dynamics of *R_1_^M1^* (open diamonds), *R_1_^M2^* (open circles), *R_1_^M3^* (open triangles). The adsorption dynamics of *M_1_* (closed diamonds), *M_2_* (closed circles), and *M_3_* (closed triangles) are shown as a reference. B) The adsorption dynamics of *M_1_^R1^* (open diamonds), *M_1_^R2^* (open circles), and *M_1_^R3^* (open triangles). The adsorption dynamics of *R_1_* (closed diamonds), *R_2_* (closed circles), and *R_3_* (closed triangles) are shown as a reference. All experiments were carried out at 24°C at an MOI of ∼0.1 with an *E. coli* cell concentration of ∼3×10^9^ cfu·ml^−1^. Adsorption data is plotted as the concentration of free phages remaining in solution normalized to the initial titer. The curves indicate trends and are not the result of a modeling equation.
(TIFF)
