# Supplementary figures and images for: Evidence That the Heterogeneity of a T4 Population Is the Result of Heritable Traits
Source: PLoS One. 2014 Dec 31;9(12):e116235. doi: 10.1371/journal.pone.0116235 (PMC4281060; doi:10.1371/journal.pone.0116235)

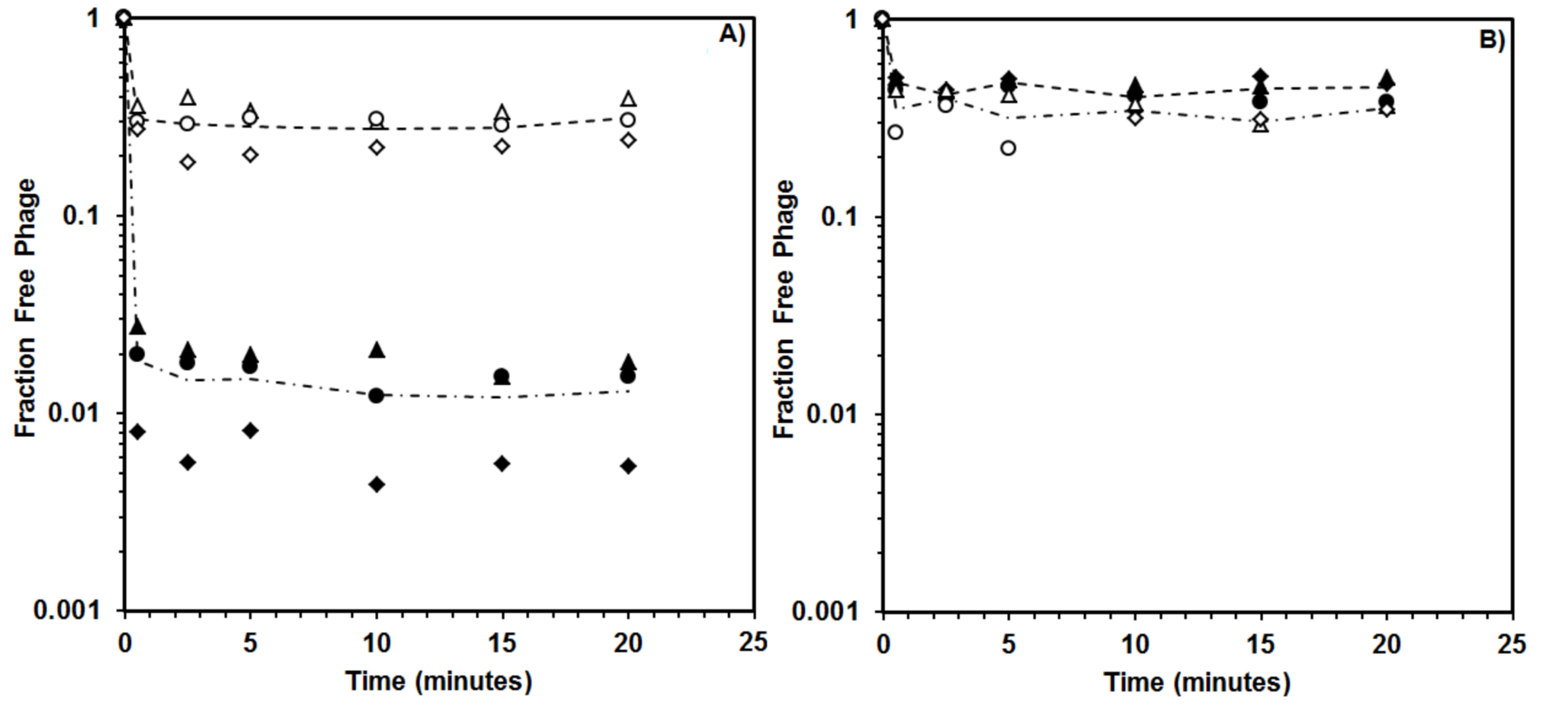

Supplement: S1 Fig — Adsorption of R1Mi and M1Ri phage stocks. A) The adsorption dynamics of R1M1 (open diamonds), R1M2 (open circles), R1M3 (open triangles). The adsorption dynamics of M1 (closed diamonds), M2 (closed circles), and M3 (closed triangles) are shown as a reference. B) The adsorption dynamics of M1R1 (open diamonds), M1R2 (open circles), and M1R3 (open triangles). The adsorption dynamics of R1 (closed diamonds), R2 (closed circles), and R3 (closed triangles) are shown as a reference. All experiments were carried out at 24°C at an MOI of ∼0.1 with an E. coli cell concentration of ∼3×10^9^ cfu·ml−1. Adsorption data is plotted as the concentration of free phages remaining in solution normalized to the initial titer. The curves indicate trends and are not the result of a modeling equation. (TIFF) [file pone.0116235.s001.tiff]
